# Treatment Selection Choices Should Not Be Based on Benefits or Costs Alone: A Head-to-Head Randomized Controlled Trial of Antiviral Drugs for Hepatitis C

**DOI:** 10.1371/journal.pone.0163945

**Published:** 2016-10-14

**Authors:** Perica Davitkov, Apoorva Krishna Chandar, Amy Hirsch, Anita Compan, Marina G. Silveira, Donald D. Anthony, Suzanne Smith, Clare Gideon, Robert A. Bonomo, Yngve Falck-Ytter

**Affiliations:** 1 Louis Stokes Cleveland VA Medical Center, Cleveland, Ohio, United States of America; 2 University Hospitals Case Medical Center, Cleveland, Ohio, United States of America; 3 Case Western Reserve University, Cleveland, Ohio, United States of America; 4 Geriatric Research Education and Clinical Center (GRECC), Louis Stokes Cleveland VA Medical Center, Cleveland, Ohio, United States of America; Yonsei University College of Medicine, REPUBLIC OF KOREA

## Abstract

**Background:**

Clinicians often face dilemmas with decisions related to formulary choices when two similar drugs are simultaneously available in the market. We studied the comparative safety, effectiveness, and treatment costs of the two first generation direct-acting antiviral agents (DAA), boceprevir and telaprevir as uncertainty existed regarding the drug of choice between these two seemingly equally Hepatitis-C treatment options.

**Methods:**

We randomly assigned 50 patients in an open-label, pragmatic randomized controlled trial (RCT) at a VA Medical Center to either boceprevir or telaprevir in combination with peginterferon and ribavirin, stratified by the presence of cirrhosis and prior treatment experience. Tolerability was assessed at each visit and reasons for discontinuation of treatment and severity of adverse events due to PI treatment were adjudicated using a blinded adjudication committee. The primary outcome was difference in tolerability between boceprevir vs. telaprevir. Secondary outcomes included viral response rates and cost-per cure achieved.

**Results:**

Higher rates of treatment discontinuations and/or severe DAA associated adverse events were seen in 10/25 (40%) patients randomized to telaprevir compared to 2/25 (8%) patients randomized to boceprevir (RR: 5; 95% CI: 1.2, 20; p<0.01). Cure rates did not appear to be significantly different between groups (telaprevir vs. boceprevir: RR 1.23; 95% CI: 0.76, 1.99; p = 0.39). On an intention-to-treat basis, total cost per cure was $44,329 for boceprevir vs. $57,115 for telaprevir. The significant side effect profile of telaprevir combined with the availability of highly efficacious second generation DAAs led to the early discontinuation of the trial.

**Conclusion:**

Telaprevir is associated with a significantly higher rate of severe adverse events leading to treatment discontinuations, hospitalizations or severe anemia and a substantially higher cost per SVR when compared to boceprevir. Real-time, point of care, pragmatic randomized controlled trials are necessary for guidance beyond just acquisition costs and to make evidence-based formulary selections when multiple effective treatments are available. (Clinicaltrials.gov registration: NCT02113631).

## Introduction

Globally/Worldwide, approximately/about/ 185 million people are infected with the Hepatitis C virus (HCV).[1, 2] In the United States, between 2.7 and 3.5 million people are believed to be infected with HCV.[3, 4] Approximately 5.4% of veterans in the United States have chronic HCV, which is more than double the estimated prevalence of HCV in the general US population.[5] Untreated HCV is likely to progress to liver cirrhosis and is strongly associated with the development of hepatocellular carcinoma.[6–8] The risk of mortality among untreated veterans with chronic HCV is nearly 25 per 1000 person years.[9] Historically, pegylated-interferon (Peg-IFN) and ribavirin (RBV) based combination therapy resulted in low cure rates (sustained virologic response [SVR]) of 30% to 45% in clinical trials.[10–12] However, results of these trials did not translate to similar findings in the clinical setting where much lower SVR rates were observed.[13] Peg-IFN/RBV combination therapy was also associated with substantial hematological, dermatological, gastrointestinal side effects, in addition to other adverse reactions, thereby greatly increasing the risk of treatment discontinuations and dose reductions.[14]

The first generation nonstructural 3/4A protease inhibitors, boceprevir and telaprevir, were approved by the United States Food and Drug Administration (FDA) in 2011 for the treatment of HCV genotype 1 (GT1) infection.[15, 16] When compared to SVR rates obtained with the standard treatment regimen of Peg-IFN with RBV (38–44%) in clinical trials, the addition of boceprevir or telaprevir demonstrated improved cure rates between 63% to 75% for GT1 patients.[17, 18] Following approval of these two direct-acting antiviral agents (DAA), clinicians largely preferred telaprevir over boceprevir based on clinical judgment of data gathered from placebo controlled phase 2 and 3 randomized controlled trials (RCT).[19, 20] The decision to use telaprevir for some clinicians might have been influenced by its shorter treatment duration (12 weeks) vs. boceprevir (24/44 weeks). Alternatively, as direct head-to-head evidence comparing the tolerability and effectiveness of these two DAAs did not exist at that time to guide clinicians, the Veterans Health Administration (VHA) decided to make boceprevir the formulary choice presumably based on an acquisition cost perspective.

Pragmatic RCTs can play an important role in facilitating decisions in selection between seemingly comparable treatment choices. The primary objective of this RCT was to compare tolerability of boceprevir vs. telaprevir in HCV treatment in treatment naïve and treatment experienced veterans. The secondary objectives were: a) to evaluate the effectiveness of boceprevir vs. telaprevir in combination with peginterferon and ribavirin; b) to examine comparative costs (drug acquisition cost plus cost of care, such as adverse effect management) per SVR achieved with the two triple therapy regimens in a Veterans’ Affairs (VA) hospital setting.

## Methods

### Study Design

This was an open-label, parallel group, randomized controlled trial. A computer generated variable (4 to 6) block randomization sequence was utilized to allocate subjects to one of two treatment groups, boceprevir or telaprevir. Allocation was concealed using serially numbered, opaque, sealed envelopes. Study subjects were stratified based on prior treatment experience and presence or absence of cirrhosis. There was blinded adjudication of key clinical outcomes. The adjudication committee was comprised of two hepatologists (YFY and MGS) who determined if adverse events (AE) leading to treatment discontinuation were related to the DAAs being studied. The committee also made decisions of treatment cessation due to virologic failure.

Reporting of this RCT is in accordance with the CONSORT 2010 statement.[21] The study was conducted in adherence with the principles of good clinical practice (GCP). This investigation was approved by the Institutional Review Board at the Cleveland VA Hospital. All patients provided written informed consent. An incentive was not offered to patients for participating in the study. All authors had access to the study data and have reviewed and approved the final manuscript as submitted (Clinicaltrials.gov registration: NCT02113631).

### Patients

Patients were recruited from the hepatitis C outpatient clinic. The trial was conducted as a “pragmatic RCT”, embedded into routine clinical care with inclusion/exclusion criteria identical to current clinical practice. Eligible patients were adult veterans (≥18 years) with HCV GT1 infection and evidence of chronic hepatitis. The presence of cirrhosis was confirmed by a liver biopsy completed within 3 years before enrollment in the study. If a liver biopsy was not available, a clinical assessment of cirrhosis risk was performed by the two independent hepatologists (YFY and MGS). Other inclusion criteria were: a platelet count ≥ 60,000/mm^3^; absolute neutrophil count ≥ 1000/mm^3^; hemoglobin ≥11 g/dL for females or ≥12 g/dL for males; serum creatinine ≤ 1.5 mg/dL; patients needed to demonstrate adequately controlled diabetes mellitus and normal or adequately controlled TSH on prescription medication. Patients with compensated liver cirrhosis were eligible to participate in the study. Patients who were previously treated with Peg-IFN/RBV and were non-responders (NR), partial responders, or relapsers were also eligible.

Exclusion criteria were based on standard prescribing information for boceprevir and telaprevir.[15, 16] These included: co-infection with Hepatitis B Virus (HBV) or Human Immunodeficiency Virus (HIV), severe unstable neuropsychiatric disorders as determined by psychological evaluation, nursing or pregnant women, patients with a malignancy diagnosed and/or treated within the past 3 years (except for localized squamous or basal cell cancers), and current alcoholism or drug addiction.

### Treatments

Boceprevir and telaprevir were administered through regular clinical care without masking (open label). All treatment doses were provided according to the standard Food and Drug Administration labeling for the two drugs. Patient compliance was monitored at regular intervals by a team comprised of a pharmacist (AH) and a nurse practitioner (AC). This trial consisted of two parallel groups: group 1—boceprevir [200 mg capsules, 800 mg TID p.o] and Peg-IFN/RBV and group 2—telaprevir (375mg capsules, 750 mg TID p.o.) and Peg-IFN/RBV.

Dosing for boceprevir (according to product labeling): HCV treatment naïve patients and relapsers received boceprevir for 24 to 44 weeks and 36 to 48 weeks respectively in addition to Peg-IFN/RBV for 28 to 48 weeks. Treatment duration was dependent on patient response after 4 weeks of treatment. Prior non-responders or patients with compensated cirrhosis received boceprevir for 44 weeks and Peg-IFN/RBV for 48 weeks.

Dosing for telaprevir (according to product labeling): HCV treatment naïve patients/relapsers received telaprevir for 12 weeks and Peg-IFN/RBV for 24 or 48 weeks. Again, treatment duration was dependent on patient response after 4 weeks of treatment. Non-responders and patients with compensated cirrhosis were prescribed telaprevir for 12 weeks and Peg-IFN/RBV for 48 weeks.

Additional therapy, dose, and mode of administration for both groups: Peginterferon alfa-2a 180 mcg s.c. every week and weight-based ribavirin 1200 mg/day p.o. divided twice daily for patients ≥75kg and 1000 mg/day p.o. divided twice daily for patients <75kg.

### Treatment outcomes

The primary endpoint of this trial was tolerability of the DAAs. Data regarding treatment tolerability and adverse events were assessed at study entry and at DAA therapy weeks 0 and 2 followed by every 4 weeks until the end of treatment as per the product labeling. All AEs, the incidence of specific AEs, and severe adverse events (SAE) leading to treatment discontinuation or hospitalization were documented and presented to the blinded adjudication committee which further decided if the AEs were protease inhibitor (PI) related.

Secondary endpoints of the study were: a) achievement of SVR24 defined as undetectable plasma HCV-RNA 24 weeks after the end of treatment; b) resource use: disaggregated data on drug acquisition cost and costs of clinical services used to treat patients and reimbursement costs based on the Medicare fee schedule and other published data were used to determine cost per SVR for both boceprevir and telaprevir.

### Statistical Analysis

This study was projected to enroll a total of 200 subjects (100:100) in groups 1 and 2 respectively. Using a power of 80% and confidence level of 95% with p-value of 0.05, to determine 20% difference in tolerability between both drugs, a sample size of 182 was determined. Accounting for losses to follow up, an increase of 10% to the sample size was projected.

Primary and secondary endpoints were analyzed using the intention-to-treat (ITT) analysis. SVR response rates were calculated using relative risk (RR) differences using chi-square test. While interim analyses were not planned, the study was stopped early after a number of serious adverse events (in particular rash with telaprevir) were increasingly reported. Resource use was prospectively recorded in disaggregated form.

## Results

### Patient Characteristics and Demographics

**Fig 1:**
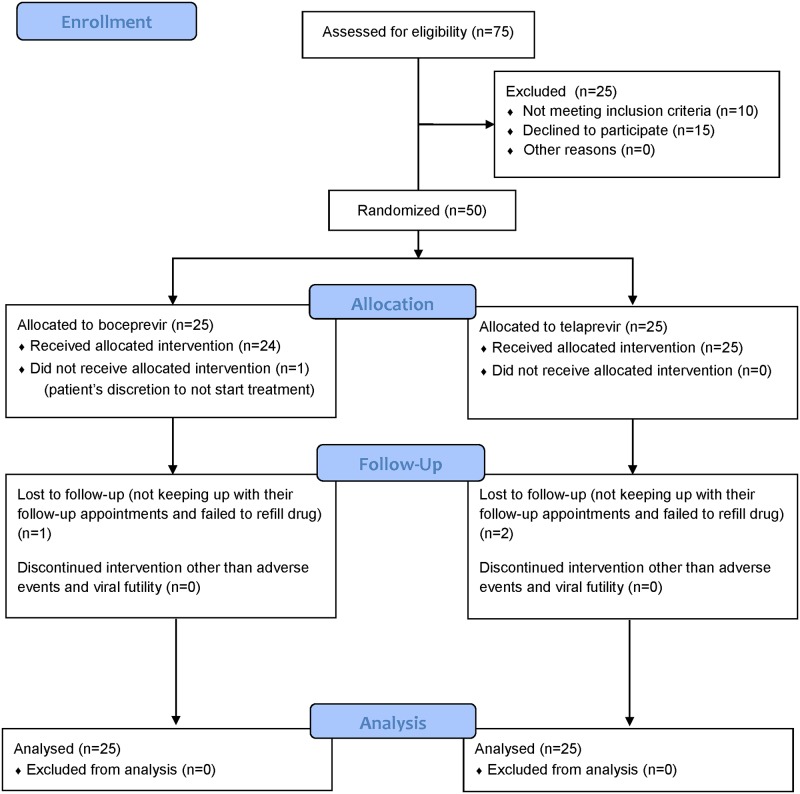
CONSORT Flow Diagram.

From September 2011 to April 2013, 50 patients were randomized in a 1:1 fashion to either boceprevir in combination with Peg-IFN/RBV or telaprevir and Peg-IFN/RBV. Of the 50 patients randomized, 3 were lost to follow-up (1 in the boceprevir group, 2 in the telaprevir group). The CONSORT study flow diagram is shown in Fig 1.

The majority of enrolled patients were men (98%) with an average age of 59 years. Forty-four percent were African American, 48% were Caucasian and 4% were Hispanic or Latino. Liver biopsy results revealed advanced fibrosis or cirrhosis (F3 and F4) in 10/50 (20%), and 2/50 (4%) patients had cirrhosis determined with clinical assessment by the two independent hepatologists. Thirty-six percent of patients were previously treated with Peg-IFN/RBV and out of these 12 (57%) were treatment failures and nine (43%) were prior treatment responder-relapsers. Patient characteristics are shown in Table 1.

**Table 1:** Demographic characteristics of patients randomized to boceprevir and telaprevir.

	Boceprevir (n = 25)	Telaprevir (n = 25)
Age [M (SD)]	59 (3.74)	59 (4.44)
Race [n (%)]		
• White	15 (60)	9 (36)
• Black	9 (36)	14 (56)
• Hispanic	1 (4)	2 (8)
Gender [n (%)]		
• Male	25 (100)	24 (96)
Advanced fibrosis (3/4) or cirrhosis [n (%)]	6 (24)	6 (24)
Treatment exposure [n (%)]		
• Naive	16 (64)	16 (64)
• Relapsers	4 (16)	5 (20)
• Non-responders	5 (20)	4 (16)

M: Mean; SD: Standard Deviation.

### Treatment tolerability and safety

Higher rates of treatment discontinuations and/or severe PI associated AEs were seen in 10 of 25 (40%) patients randomized to telaprevir compared to 2 of 25 (8%) patients randomized to boceprevir (RR: 5; 95% CI: 1.2, 20; p<0.01). Eight patients discontinued telaprevir treatment early due to toxicity (mostly due to rashes); one patient required hospitalization due to progressive maculopapular rash, and another developed severe anemia (Hb ≤8 g/dL). Of the two patients on boceprevir who stopped treatment early, one experienced a severe rash and the other had severe dysgeusia.

Four patients (3 on boceprevir and 1 on telaprevir) discontinued treatment early due to adverse events felt most likely related to Peg-IFN/RBV. Two patients (one in each treatment group) experienced mood disturbances while two patients on boceprevir experienced nausea, vomiting and flu-like symptoms. Treatment tolerability data are presented in Table 2.

**Table 2:** Tolerability of boceprevir and telaprevir.

	Boceprevir (n = 25)	Telaprevir (n = 25)	P value
Treatment-emergent AEs due to PI leading to treatment discontinuation [n (%)]			
• Toxicity*	2 (8)	8 (32)	0.04
• Hospitalization	0 (0)	1 (4)	0.77
• Severe anemia (<8 g/dL)	0 (0)	1 (4)	0.77
• Total	2 (8)	10 (40)	<0.01
AEs due to Peg-IFN/RBV leading to treatment discontinuation [n (%)]			
• Mood disturbances	1 (4)	1 (4)	>0.99
• Nausea, vomiting and flu-like symptoms	2 (8)	0 (0)	0.43

*Toxicity: Severe rash, rectal burning/ pruritus, and dysguesia, PI: Protease Inhibitor; AE: Adverse Event; Peg-IFN: Pegylated Interferon; RBV: Ribavirin.

Based on the overall poor tolerability of the treatment regimens, the investigators decided to stop the trial early. Patients who tolerated telaprevir poorly were given an option to either switch to Boceprevir, which was associated with fewer side effects, or continue without treatment until the VA formulary approved the second generation DAAs.

### Treatment effectiveness

Of the 25 patients randomized to receive boceprevir, 11 patients (44%) achieved SVR24 and of the 25 patients randomized to telaprevir, 12 patients achieved SVR24 (48%). SVR24 rates by treatment stratification are presented in Table 3.

**Table 3:** SVR24 rates based on treatment exposure to boceprevir and telaprevir.

	Boceprevir (n = 25)	Telaprevir (n = 25)	RR (95%CI)	P value
Treatment naïve (n = 32; 25% with cirrhosis)	7/16	8/16	0.9 (0.5–1.6)	0.35
Treatment experienced [partial viral responders, non-responders and relapsers] (n = 18; 22% with cirrhosis)	4/9	4/9	1 (0.3–3.1)	0.5
Total (n = 50; 24% with cirrhosis)	11/25	12/25	1.07 (0.6–1.8)	0.39

SVR: sustained virologic response; RR: Relative Risk.

Two patients on boceprevir (8%) and 1 patient on telaprevir (4%) experienced viral relapse between weeks 4 and 12 after completion of treatment. According to futility criteria as per FDA package label, 6/25 in boceprevir and 0/25 in telaprevir were considered to have met the futility criteria due to viral breakthrough or non-response.

### Resource use

Patients in the boceprevir group had on average 13 outpatient visits related to their HCV treatment vs. 9 outpatient visits for those in the telaprevir group (mostly due to the high percentage of early terminations associated with telaprevir). Mean cost of standard triple therapy per patient with boceprevir and Peg-IFN/RBV and routine care was $18,670 ($7,958 –$32,877) in comparison to $26,962 ($363 –$30,505) per patient in the telaprevir and Peg-IFN/RBV group.

Additionally, adverse event management (additional outpatient visits, emergency department [ED] evaluations, hospitalizations and additional medications, such as growth factors) was $14,801 vs. $24,594 for boceprevir and telaprevir respectively. In the boceprevir group, 6 patients received epoetin-α, 1 received filgrastim, 4 patients required additional outpatient visits and 1 patient required an ED evaluation. In the telaprevir group, 7 patients received epoetin-α, 1 received filgrastim, 3 patients required additional outpatient visits, 2 patients needed an ED evaluation and 2 patients were briefly hospitalized. On an ITT basis, total cost per SVR achieved was $44,329 for boceprevir vs. $57,115 for telaprevir (Table 4).

**Table 4:** Resource use associated with boceprevir and telaprevir.

	Boceprevir (n = 25)	Telaprevir (n = 25)
**Routine care**		
• Cost for Routine care NP/physician/PharmD/MH visits	$31,823.00	$25,168.00
• Routine drugs cost (PI/Peg-IFN/RBV)	$440,997.78	$648,884.00
• Total routine care cost per person	$18,912.83	$26,962.08
**Additional care**		
• Cost for extra evaluation due to AE*	$2,260.00	$11,330.00
• Drugs used for treatment of AE (Epo+Fil)	$12,541.00	$13,264.00
• Cost of adverse events per study arm	$14,801.00	$24,594.00
**Total treatment cost per SVR**	$44,329.25	$57,115.17

*Outpatient evaluation by nurse practitioner, pharmacist, mental health provider, emergency department evaluations, hospitalizations

Epo: epoetin-α; Fil: filgrastim; PI: Protease Inhibitor; AE: Adverse Event; MH: Mental Health; NP: Nurse Practitioner; PharmD: Clinical Pharmacist; Peg-IFN: Pegylated Interferon; RBV: Ribavirin; SVR: Sustained virologic response.

## Discussion

Compared to boceprevir, use of telaprevir in standard triple antiviral regimens within a Midwestern VA patient population was associated with a significantly higher rate of severe adverse events (treatment toxicity) leading to treatment discontinuations. Treatment efficacy rates were not significantly different between the two treatment groups, but both telaprevir and boceprevir showed substantially lower viral cure rates than observed in the registration trials. Cost of treatment per cure was substantially higher for telaprevir when compared to boceprevir.

The approval of two novel protease inhibitors in 2011 sparked an interest in hepatitis C treatment. Given the lack of evidence-based guidelines concerning hepatitis C treatment when these drugs were approved, clinicians preferred using telaprevir based triple therapy, presumably given its shorter treatment duration and a perceived better response rate since real-world treatment experience reports were lacking.[19, 20] In contrast, boceprevir was the formulary choice in VHA based on its competitive pricing. Both treatment selection approaches should have been classified as preliminary as they either focused solely on viral cure and ignored the balance between benefits and harms or made drug utilization choices based on acquisition cost rather than comparative cost per cure.Observational data which subsequently emerged, such as those from the CUPIC study group in 2013, showed an increased incidence in SAEs including increased deaths with telaprevir when compared to boceprevir, despite higher cure rates with telaprevir.[22] Yet, such observational registry data, while useful, rarely provides reliable comparative effectiveness estimates, mainly due to uncontrollable selection bias.

Pragmatic RCTs are a methodologically rigorous way of studying comparative effectiveness in an unselected population.[23] Availability of such head-to-head RCT data become even more important when the side-effect profiles of the drugs in question are questionable. Although boceprevir compared to telaprevir appeared attractive from an acquisition cost perspective, implementing such guidance without comparative evidence for effectiveness and harms, especially given the high cost of late-stage complications, may not always lead to the desired and predicted cost savings. Initial drug selection for formulary inclusion based on drug acquisition cost can be a reasonable strategy; however, subsequent comparative effectiveness trials provide an ideal strategy to ensure long-term cost-effective prescribing, particularly when drug unit costs are high.

Most (~93%) veterans with chronic HCV in Veterans Affairs (VA) care are 50 years or older, and more than a third are older than 60 years. As a result, hepatitis C is an important disease of aging; especially since older patients typically have lower tolerability thresholds to antivirals. Therefore, we felt it was necessary to collect reliable, low risk-of-bias data about HCV therapies made available following regulatory approval in order to select the more tolerable and effective option to prevent the development of complications of untreated HCV infection.

Our analysis has several limitations. Given the adverse effects associated with the drugs, enrollment in the study was terminated early. Since the expected sample size was not achieved, our study was underpowered to detect important differences in treatment efficacy. However, despite a smaller sample size, a statistically and clinically significant difference in DAA tolerability was evident, which was our primary outcome measure. Second, our study was also underpowered to detect any differences among important sub-populations, such as patients with cirrhosis or women. Additionally, the FDA approved two new DAAs, namely sofosbuvir and simprevir in the fall of 2013, both of which showed better efficacy and a more favorable side effect profile than the first generation DAAs. Given the availability of these newer DAAs and the impending approval of these drugs for use by the VHA formulary, we felt that it was unnecessary to continue treating patients with either telaprevir or boceprevir. Nevertheless, our trial underscores the need for pragmatic RCTs as they help us get a better, real-world understanding of the comparative efficacy and tolerability of drugs that have hitherto only been compared against placebo.

Pragmatic RCTs in the context of the newer generation of hepatitis C therapies merits discussion. These newer antiviral treatments achieve high SVR rates and are generally well tolerated in registration trials[24–29] although real life data on adverse events are sparse. Despite the universally high SVR rate (>90%) of newer antiviral combination therapies (such as sofosbuvir/ledipasvir and ombitasvir/paritaprevir/ritonavir/dasabuvir), small differences in tolerability or adverse events can significantly impact tolerance and thus SVR rates seen in real-life treatment results. These drugs typically cost between $63,000 to $94,500 for 8-12-week regimens.[30] When treatment costs are high, comparative effectiveness data become important. While cost-simulation data can provide indirect estimates of cost-effectiveness,[31] they are limited in their use because of lack of real-world cost-effectiveness data. Non-randomized studies have also been performed, but these also suffer from the inability to provide real-time comparative effectiveness data and have their own inherent biases because of their observational design.[32, 33] Pragmatic RCTs can provide real-time, comparative effectiveness and resource use data. Particularly when treatment costs are high as in the case of these newer antiviral combination regimens, even small differences in treatment efficacy and/or adverse events leading to treatment discontinuation will lead to significant differences in cost per SVR achieved.

## Conclusion

Head-to-head trials, particularly comprising of a sicker and aging population can be particularly useful in guiding hepatitis C treatment choices. Based on our findings and the availability of newer DAAs, we discontinued the initiation of new treatment regiments with telaprevir. Real-time, point of care, pragmatic randomized controlled trials are necessary to make evidence-based treatment selections when multiple effective treatments are available.

## Supporting Information

S1 File CONSORT 2010 checklist of information to include when reporting a randomised trial. (DOC)

S2 File Study Protocol. (DOCX)

## Abbreviations

AE: Adverse Event
CUPIC: Compassionate Use of Protease Inhibitors in Viral C Cirrhosis
DAA: Direct-acting Antiviral Agents
ED: Emergency Department
FDA: US Food and Drug Administration
GCP: Good Clinical Practice
GT: Genotype
HBV: Hepatitis B virus
HCV: Hepatitis C Virus
HIV: Human Immunodeficiency Virus
IFN: Interferon
ITT: Intention-to-treat
NR: Non-responder
PegIFN: Pegylated Interferon
PI: Protease Inhibitor
RBV: Ribavirin
RCT: Randomized Controlled Trial
RR: Relative Risk
SAE: Severe Adverse Event
SVR: Sustained Virologic Response
TSH: Thyroid-Stimulating Hormone
VA: Veterans Affairs
VHA: Veterans Health Administration
