# Supplementary material for: Treatment Selection Choices Should Not Be Based on Benefits or Costs Alone: A Head-to-Head Randomized Controlled Trial of Antiviral Drugs for Hepatitis C
Source: PLoS One. 2016 Oct 14;11(10):e0163945. doi: 10.1371/journal.pone.0163945 (PMC5065164; doi:10.1371/journal.pone.0163945)
Supplement: S2 File — (DOCX) [file pone.0163945.s002.docx]

**Study Protocol**

**Title:** Comparative effectiveness and tolerability of boceprevir vs. telaprevir and re-assessment of treatment duration in patients with chronic hepatitis C.

**Duration of Study:** Approximately 48 months

**Objectives:**

**Primary Objective:** The primary objective is to study the comparative tolerability of boceprevir vs. telaprevir in HCV treatment in treatment naïve and treatment experienced veterans.

**Secondary Objectives:**

1. To study the comparative effectiveness of boceprevir vs. telaprevir in HCV treatment in treatment naïve and treatment experienced veterans.
2. Other secondary objectives include recording of differences in resource use, such as direct costs (e.g., drug acquisition costs) and other indirect cost (e.g., staff utilization etc.)

**Background:**

An estimated 170 million people worldwide are infected with hepatitis C and 4 million of them are in the United States. Around 85% of patients who become infected with hepatitis C fail to clear the virus and become chronic carriers. Among these individuals, 5%to 20% are reported to develop cirrhosis over a period of approximately 20 to 25. For almost a decade, the standard of care (SOC) in patients with chronic hepatitis C has consisted of a 24–48 week course of PEG-IFN-α2a or PEG-IFN-α2b in combination with ribavirin. This therapy leads to a sustained virologic response (SVR) in 42–52% for HCV genotype 1, 65–85% for HCV genotypes 4, 5 or 6 and 76–82% of individuals infected with HCV genotypes 2 or 3.

During the past 10 years, intensive efforts have been made to develop different compounds with antiviral activities against HCV genotype 1, which are referred to as direct acting antiviral agents (DAAs). The clinical development program of the two HCV nonstructural (NS) 3/4A protease inhibitors boceprevir and telaprevir has now been completed. Few reviews, including narrative reviews and randomized trials have been published so far. Their overall conclusion is that adding one of the protease inhibitors to the standard treatment increases the number of SVRs up to 75% in patients who relapse and 40 – 52 % in patients who didn’t respond.

**Scientific Rationale:**

Both of these drugs have recently been approved by the FDA and are now beginning to be used at the Cleveland VA for the treatment in Hepatitis C, genotype 1. However, the effectiveness and safety of these protease inhibitors within the VA population remain undefined. Since the vast majority of the Hepatitis C patient population at the VA is genotype 1 (approx. 80%), results from this trial will have a significant impact on our treatment recommendations for this population.

The prevalence of HCV infection in VA population is ~60% in persons between 50-60 years old, which means that in 3-5 years, a larger proportion of the VA population with HCV infection will be over 65 years. Furthermore, discontinuation rate in the studies with highly selected population is around 20%; however, we expect this rate to be significantly higher in our population. In addition, older age, multiple co-morbidities and complex drug regimens can have a major impact on treatment effectiveness.

**Methodology:**

Boceprevir and telaprevir (in combination with peg-interferon alpha 2a and ribavirin [PR]) will be randomly assigned in a 1:1 using a variable block randomization sequence. Boceprevir or telaprevir will be administered through regular clinical care without masking (open label) in previously untreated as well as treatment experienced patients with HCV genotype 1, to be conducted in conformance with Good Clinical Practice (GCP).

This trial consists of two parallel groups: group 1- Boceprevir + PR; group 2 – Telaprevir + PR;

**Type of Blinding:** open-label; however, blinded adjudication of key outcomes, such as adverse events leading to treatment discontinuation, and decisions on treatment cessation due to viral failure will be carried out.

**Allocation concealment:** serially numbered, opaque, sealed envelopes; variable block randomization.

**Sample Size (Including Ratio of Subjects Assigned to Treatments)/Power:** This study is projected to enroll a total of 200 subjects (100:100) in group 1 and 2 respectively.

Given a power of 80% and confidence level of 95% with p-value of 0.05, to determine a 20% difference in tolerability between both drugs, a sample size of 182 is required. Accounting for losses to follow-up, an increase of 10% to the sample size is projected.

**Randomization:** Subjects will be randomized in the two treatment arms in a 1:1 ratio. Randomization will be based on a computer-generated random code.

**Stratification:** Eligible patients will be stratified into 4 groups (treatment naive, treatment experienced; with and without compensated cirrhosis).

**Diagnosis and Criteria for Inclusion:** Adult subjects with CHC HCV genotype 1.

The subject must meet ALL criteria listed below for entry:

**Inclusion Criteria:**

1. At least 18 years of age
2. Have HCV genotype 1 infection and evidence of chronic hepatitis, as confirmed by a liver biopsy completed within three years prior to enrollment in the study (standard of therapy). Patients with compensated liver cirrhosis will be eligible. Patients who have previously been treated under standard of care (Peg-IFN, Ribavrin) and were non-responders, partial responders, or relapsers will also be eligible.
3. Platelet count ≥ 60,000/mm3; absolute neutrophil count ≥ 1000/mm3 ; hemoglobin ≥11.0 g/dL for females or ≥12.0 g/dL for males; serum creatinine </= 1.5 mg/dL
4. Adequately controlled DM
5. Normal or adequately controlled TSH on prescription medication
6. All other clinical laboratory values within normal limits, unless judged not clinically significant by the investigator
7. Capable of understanding instructions, adhering to study schedules and requirements, and willing to provided informed consent

**Exclusion Criteria:**

1. Positive HIV or HbsAg serology
2. Severe psychiatric or neuropsychiatric disorders including, but not limited to uncontrolled severe depression, history of suicidal ideations or suicide attempt(s), as determinate by psychological evaluation
3. Chronic hepatic diseases other than hepatitis C
4. Organ or bone marrow transplant
5. Chronic (greater than 30 days) use of immunosuppressive medications including steroids in doses equivalent to 10 mg of prednisone or higher, 30 days prior to and anytime during the course of the study
6. Female patients who are breast-feeding or have a positive pregnancy test at any time during the study
7. Patients who have had a malignancy diagnosed and/or treated within the past 3 years, except for localized squamous or basal cell cancers treated by local excision
8. Current alcoholism or drug addiction

**Product, Dose, Mode of Administration:** Boceprevir (Victrelis) 200 mg capsules, 800 mg TID po vs. Telaprevir (Incivek) 375mg capsules, 750 mg TID po.

**Additional Therapy, Dose, Mode of Administration for both groups:** Peginterferon alfa-2a 180 mcg s.c., weight-based ribavirin 1200 mg/day po divided twice daily (BID) (patient weight >/= 75kg) and 1000 mg/day po divided twice daily (BID) (patient weight < 75kg). Peginterferon alfa-2b will not be permitted as it is a) less efficacious, b) to keep the co-antiviral treatment comparable and standardized.

**Duration of Treatment:**

Group 1 – Boceprevir (according to product labeling):

- HCV treatment naïve patients: boceprevir for 24 to 44 weeks; PR for 28 to 48 weeks depending on RVR achievement at week 4 of protease inhibitor treatment;
- Relapsers and partial responders: boceprevir for 36 weeks; PR for 36 to 48 weeks depending on RVR achievement at week 4 of protease inhibitor treatment;
- Prior non-responders, or patients with compensated cirrhosis: boceprevir for 44 weeks; PR for 48 weeks

Group 2 – Telaprevir (according to product labeling):

- HCV treatment naïve patients/relapsers: telaprevir for 12 weeks, PR for 24 or 48 weeks depending on RVR achievement at week 4 of protease inhibitor treatment;
- Non-responders and patients with compensated cirrhosis: telaprevir for 12 weeks, PR for 48 weeks

**Criteria for Evaluation:**

Safety and effectiveness assessments will be conducted at study entry, Protease inhibitor therapy week 0, 4, 8, 12, 16, 20, 24, 28, 32, 36, 40, 44 and 48 according to product labeling, if applicable. These will include blood tests, history and complete physical exams. SVR will be assessed at 24 weeks after treatment has been completed.

**Statistical Methods:**

Descriptive statistics will be used for rates (SVR, RVR, end of treatment response, adherence). The primary and secondary endpoints will be determined on an intention-to-treat (ITT) basis. SVR response rates will be analyzed using chi-square test. Equivalency of response rates for the secondary analysis (reduction in treatment length) will be defined as SVR rates that are within the 10% boundaries compared to standard of care. No interim analyses will be performed. As the study is carried out within the regular hepatitis clinic in an unblinded fashion with FDA approved drugs, no data and safety monitoring board will be necessary. Resource use will be prospectively recorded in disaggregated form. Formal cost-effectiveness analysis will be carried out at the end of the study.
